# Are potentially clinically meaningful benefits misinterpreted in cardiovascular randomized trials? A systematic examination of statistical significance, clinical significance, and authors’ conclusions

**DOI:** 10.1186/s12916-017-0821-9

**Published:** 2017-03-20

**Authors:** G. Michael Allan, Caitlin R. Finley, James McCormack, Vivek Kumar, Simon Kwong, Emelie Braschi, Christina Korownyk, Michael R. Kolber, Adriennne J. Lindblad, Oksana Babenko, Scott Garrison

**Affiliations:** 1 Evidence-Based Medicine, Department of Family Medicine - Research Program, University of Alberta, 6-10 University Terrace, Edmonton, AB T6G 2T4 Canada; 2 Faculty of Pharmaceutical Sciences, University of British Columbia, Vancouver, British Columbia Canada; 3 Family Medicine, McGill University, Montreal, QC Canada; 4 Medical Education, Department of Family Medicine, University of Alberta, Edmonton, AB Canada

**Keywords:** Cardiovascular, Randomized controlled trials, Statistical significance, Clinical significance, Confidence intervals, Conclusions

## Abstract

**Background:**

While journals and reporting guidelines recommend the presentation of confidence intervals, many authors adhere strictly to statistically significant testing. Our objective was to determine what proportions of not statistically significant (NSS) cardiovascular trials include potentially clinically meaningful effects in primary outcomes and if these are associated with authors’ conclusions.

**Methods:**

Cardiovascular studies published in six high-impact journals between 1 January 2010 and 31 December 2014 were identified via PubMed. Two independent reviewers selected trials with major adverse cardiovascular events (stroke, myocardial infarction, or cardiovascular death) as primary outcomes and extracted data on trial characteristics, quality, and primary outcome. Potentially clinically meaningful effects were defined broadly as a relative risk point estimate ≤0.94 (based on the effects of ezetimibe) and/or a lower confidence interval ≤0.75 (based on the effects of statins).

**Results:**

We identified 127 randomized trial comparisons from 3200 articles. The primary outcomes were statistically significant (SS) favoring treatment in 21% (27/127), NSS in 72% (92/127), and SS favoring control in 6% (8/127). In 61% of NSS trials (56/92), the point estimate and/or lower confidence interval included potentially meaningful effects. Both point estimate and confidence interval included potentially meaningful effects in 67% of trials (12/18) in which authors’ concluded that treatment was superior, in 28% (16/58) with a neutral conclusion, and in 6% (1/16) in which authors’ concluded that control was superior. In a sensitivity analysis, 26% of NSS trials would include potential meaningful effects with relative risk thresholds of point estimate ≤0.85 and/or a lower confidence interval ≤0.65.

**Conclusions:**

Point estimates and/or confidence intervals included potentially clinically meaningful effects in up to 61% of NSS cardiovascular trials. Authors’ conclusions often reflect potentially meaningful results of NSS cardiovascular trials. Given the frequency of potentially clinical meaningful effects in NSS trials, authors should be encouraged to continue to look beyond significance testing to a broader interpretation of trial results.

**Electronic supplementary material:**

The online version of this article (doi:10.1186/s12916-017-0821-9) contains supplementary material, which is available to authorized users.

## Background

The preferred reporting of clinical outcomes in randomized controlled trials (RCTs) is described in the Consolidated Standards of Reporting Trials (CONSORT) statement [1]. Within CONSORT the use of confidence intervals is emphasized in preference to *p*-values. Confidence intervals describe the precision of the estimate and “are especially valuable in relation to differences that do not meet conventional statistical significance, for which they often indicate that the result does not rule out an important clinical difference” [1]. Editorials dating back almost 40 years have encouraged authors to use confidence intervals to describe the results of their studies rather than simply reporting the findings as statistically significant or not [2–4]. Despite this, the use of *p*-values in published articles remains approximately seven times more common than confidence intervals [5]. Furthermore, confidence intervals are often used in a manner similar to *p*-values, to dichotomize outcomes as statistically significant (SS) or not. We have previously written about three important clinical controversies resulting from this dichotomous activity [6].

Interpretation of trial results when primary outcomes are not statistically significant (NSS) is challenging. In particular, it can be difficult putting the potential clinical relevance of the NSS effect and confidence intervals in context of the entire study results. Boutron and colleagues demonstrated that authors often place a favorable “spin” (positive portrayal) on trial results when the primary outcome is NSS [7]. Such spin occurred in 58% of abstract conclusions, 50% of main text conclusions, and 18% of titles. Others have similarly reported spin in RCTs evaluating wound care [8] and surgical modalities [9, 10]. Although promotion of results may be common in NSS trial reporting, the evaluation assumes that NSS results demonstrate no potentially clinically meaningful effect.

For these reasons we examined the primary outcomes and conclusions of RCTs in six major medical journals. We had two primary questions: (1) How often do the point estimates and confidence intervals of the primary outcome of NSS and SS trials include potentially clinically meaningful effects? and (2) Are the authors’ conclusions in the abstract of NSS trials influenced by potentially clinically meaningful point estimates and confidence intervals? We focused specifically on cardiovascular trials with major adverse cardiovascular events (MACE) because these are established, objective, patient-oriented outcomes that overlap between trials. Additionally, in large cardiovascular trials with hard clinical endpoints, statistical significance can be difficult to attain but the results have high clinical relevance. We hypothesized that authors of cardiovascular trials may discount potentially clinically meaningful effects identified in the confidence intervals and/or point estimates when the results are NSS.

## Methods

We followed the basic approach described in PRISMA [11] because there is no agreed on methodology for this type of study.

### Eligibility and information sources

We included all cardiovascular RCTs of superiority design that evaluated preventive or interventional therapies regardless of the nature of the interventions – including medication, surgery, models of care, and lifestyle change. All comparators were valid, including placebo, active control, and no intervention. The primary outcome had to include at least one MACE: myocardial infarction, stroke, or cardiovascular death. We used PubMed to identify relevant trials from five high-impact general medical journals and one high-impact specialty journal: *New England Journal of Medicine* (*N Engl J Med*), *Lancet*, *Journal of the American Medical Association* (*JAMA*), *British Medical Journal* (*BMJ*), *Annals of Internal Medicine* (*Ann Intern Med*), and *Circulation*.

### Study search and selection

Between 17 March and 14 April 2015, we searched PubMed for papers using the full journal title (and abbreviation, if present) with PubMed limits for RCTs and date (1 January 2010 to 31 December 2014). In the case of *Circulation*, the term circulation could relate to medical/physiologic issues in addition to the journal, so we restricted the search field to “Journal”. For the other five journals we did not apply any search restrictions in order to minimize the unlikely chance of missing relevant articles. For each journal, two authors (from VK, SK, EB, and GMA) independently evaluated and selected studies for inclusion. We excluded studies of subgroups, re-analyses, and studies that were either extensions or follow-ups from previously published trials to avoid including the same data more than once. We also excluded non-inferiority designed studies because authors’ interpretations and conclusions of non-inferiority results are broader, and this would add complexity to our interpretation of abstract conclusions. Disagreements for inclusion were resolved by consensus.

### Data extraction and management

Two authors (CF with VK or SK) independently extracted data from the trials. Disagreement was resolved with consensus or third-party review (GMA).

Data extraction on study characteristics included citation, type of intervention and control, primary versus secondary prevention population, mean age in study, and percentage of males studied. Data on traditional risk of bias included allocation concealment, blinding, analysis (intention to treat or per protocol), and withdrawals. We also collected data on funding, and whether the trial was stopped early (if so, why) or extended. Data related to the primary outcome included the clinical endpoint, number of subjects in each study arm, number with the outcomes in each group, point estimate, confidence intervals, and *p*-values.

To evaluate the authors’ conclusions, the abstract conclusion was rated using a method derived from Als-Nielsen and colleague’s technique [12]. We condensed the score from six to three possible conclusions: treatment superior, neutral, or control superior.

### Assessing potentially meaningful effects

To assess if the primary outcome of an NSS trial included potentially meaningful effects, we focused on the point estimate and lower confidence interval. The margins of potentially clinically meaningful effect are undoubtedly debatable. Over 20 years ago, authors suggested that potentially clinically meaningful effects could be 25% or 50% relative risk reductions [13]. More recently, trials showing a relative risk reduction of 6% for ezetimibe [14] and 14% for empagliflozin [15] have been greeted with enthusiasm [16, 17]. We selected our margins of potentially meaningful effect liberally to be broad and inclusive, thereby ruling out what is likely not a clinically meaningful effect. We decided that the smallest potentially clinically meaningful effect was a 6% relative risk reduction or a 0.94 relative risk, as reported by the IMPROVE-IT trial for ezetimibe [14]. For lower confidence intervals to include potentially meaningful effects, we selected a 25% relative risk reduction or 0.75 relative risk described in meta-analyses of statin trials [18], an established clinical therapy.

### Analysis of results

Study characteristics and potential biases are presented descriptively. Relative effect estimates including relative risks, hazard ratios, rate ratios, and odds ratios were used for primary analysis. If not provided, relative risks and 95% confidence intervals were calculated.

Trials were initially categorized into three groups based on the statistical testing of the primary outcome: SS trials favoring control, SS trials favoring treatment, and NSS trials. Statistical significance was determined by hypothesis testing via the *p*-value first and, if not available, we determined if the confidence interval excluded 1 (the line of no-effect).

To analyze and describe the results, the primary outcomes for all RCTs were presented on a forest plot with the potentially clinically meaningful thresholds for point estimate (≤0.94) and confidence interval (≤0.75) indicated. We categorized NSS trials as having (1) both the lower confidence interval and point estimate include potentially meaningful effects; (2) either the lower confidence interval or point estimate include a potentially meaningful effect; or (3) neither the lower confidence interval nor point estimate include a potentially meaningful effect. Among NSS trials, results were further stratified according to authors’ conclusions.

We used chi-square and independent samples median test to examine if selected factors were associated with authors’ conclusions in NSS trials. Factors compared included type of control used in the trials, funding (industry, public, or mixed), point estimates, and lower confidence intervals.

### Sensitivity analyses

We performed sensitivity analyses to examine the effect of some key variables on the proportion of NSS trials with potentially clinically meaningful effect. Because smaller trials may be expected to have broader confidence intervals, we performed an analysis of trials with <2000 patient-years and those with ≥2000 patient-years. Because primary prevention trials will have smaller absolute benefits for a given relative benefit, we performed an analysis of primary versus secondary prevention trials.

To determine how sensitive the results were to the threshold of potential clinically meaningful effects, we increased the potentially meaningful relative risk reduction threshold for point estimates to ≥15% (or ≤0.85 relative risk) and for lower confidence intervals to ≥35% (or ≤0.65 relative risk).

## Results

### Study inclusion and characteristics

**Fig. 1:**
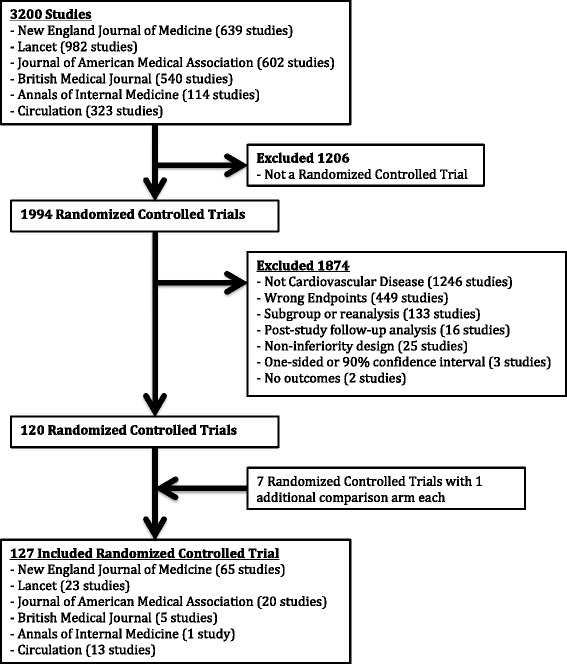
Study flow

The flow of study exclusion and inclusion is detailed in Fig. 1. Of the original 3200 studies identified in our search, 127 RCTs met inclusion criteria. Agreement for study selection was 97% and for data extraction was 93%. General characteristics and risk of bias of included studies are outlined in Table 1 and the full list of the studies is included in Additional file 1. Secondary prevention trials (85%, 108/127) and community-based trials (58%, 74/127) were most common. The primary outcome included a range of one to ten combined outcomes (median, three) with myocardial infarction (80%), stroke (65%), and cardiovascular death (50%) being the most common. Overall, study quality was good: for example, 77% (98/127) described allocation concealment and 94% (119/127) performed intention-to-treat analysis. Most trials (75%, 95/127) were completed as planned but 22% (28/127) were stopped early for varying reasons (usually harm or futility) and 3% (4/127) were extended.

**Table 1:** Study characteristics and risk of bias of the 127 included randomized controlled trials

**Study characteristics**	
Journal, n (%)	
*New England Journal of Medicine*	65 (51)
*Lancet*	23 (18)
*Journal of the American Medical Association*	20 (16)
*British Medical Journal*	5 (4)
*Annals of Internal Medicine*	1 (1)
*Circulation*	13 (10)
Setting, n	
Community	74 (58)
Hospital	53 (42)
Primary or secondary prevention, n	
Primary	19 (15)
Secondary	108 (85)
Experimental interventional, n	
Medication	65 (51)
Surgery	32 (25)
Models of care	11 (9)
Vitamin/supplement	9 (7)
Lifestyle	4 (3)
Diagnostics/other*	6 (5)
**Patient characteristics**	
Median age (interquartile range), years	63.8 (61.5–66.5)
Percent males (interquartile range)	72.0 (60.4–78.0)
Study size and duration	
Median study size (interquartile range)	3020 (1319–8521)
Median study duration (interquartile range), months	24.0 (8.3–45.3)
Primary outcome included (median 3, range 1–10), n (%)	
Myocardial infarction	101 (80)
Stroke	83 (65)
Cardiovascular death	64 (50)
Overall death	51 (40)
Revascularization	31 (25)
Heart failure	22 (17)
Other^a^	37 (29)
**Risk of bias, n (%)**	
Planned trial duration	
Completed as planned	95 (75)
Extended	4 (3)
Stopped for benefit	8 (6)
Stopped for harm	10 (8)
Stopped for futility	9 (7)
Stopped for financial reasons	1 (1)
Allocation concealment	
Yes	98 (77)
Unclear/no	29 (23)
Blinding	
Double	65 (51)
Single	13 (10)
None	49 (39)
Analysis	
Intention to treat	119 (94)
Modified intention to treat	7 (6)
Per protocol	1 (1)
Sample size estimation	
Estimation attained	83 (65)
Estimation missed	38 (30)
No estimation given	6 (5)
Withdrawal	
Number provided	115 (91)
Median (interquartile range)	2.3 (0.5–7.0)
Funding	
Industry	52 (41)
Mixed	46 (36)
Public	28 (22)
Not described	1 (1)

*Examples of other include stem cells and continuous positive airway pressure

^a^Other includes angina, thromboembolism, stent failure, cardiac arrest, renal outcomes, shock, peripheral vascular event, bleeding, arrhythmia, pericardial tamponade, respiratory failure, severe left ventricular dysfunction requiring mechanical support, hypertension, and/or aortic insufficiency

### Statistical significance of primary outcome and conclusions

**Fig. 2:**
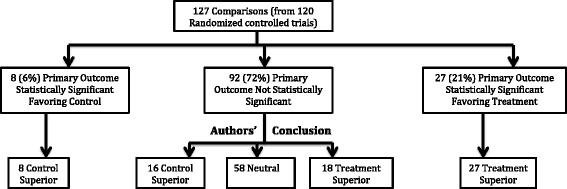
Flow of trial primary outcome including presence of statistical significance and abstract conclusion

Figure 2 outlines the flow of study outcomes with categorization by statistical significance for the primary outcome and authors’ conclusions. The primary outcome was SS favoring treatment in 21% of trials (27/127) and all concluded treatment was superior. The primary outcome was NSS in 72% of trials (92/127), of which 63% (58/92) had a neutral conclusion, 20% (18/92) concluded treatment was superior, and 17% (16/92) concluded control was superior. The primary outcome was SS favoring control in 6% (8/127) and all concluded control was superior.

### Potentially clinically meaningful effects by statistical significance

**Fig. 3:**
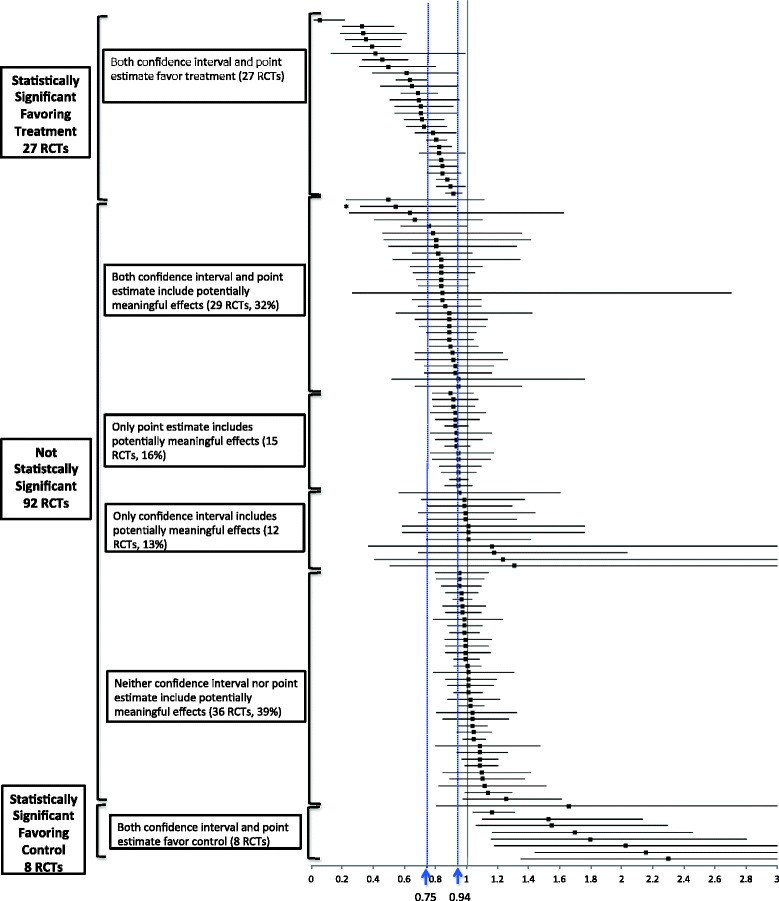
Forest plot of trial primary outcomes organized by statistically significant testing and if potentially clinical meaningful effects are indicated by the point estimates (≤0.94) and/or the confidence intervals (≤0.75). *RCT* randomized controlled trial. * Not statistically significant as *p*-value adjusted for multiple comparisons

Figure 3 provides the forest plot of all primary outcomes organized by statistical significance and if the confidence intervals and/or point estimates included potentially meaningful effects. Careful inspection of the forest plot reveals that a number of the NSS trials had lower confidence intervals and point estimates that appear similar to the effects in many of the SS trials. Among NSS trials, in 32% (29/92) both the lower confidence interval and point estimate included potentially meaningful effects, while in 29% (27/92) only one of the two included a potentially meaningful effect. Neither the lower confidence interval nor point estimate included potentially meaningful effects in 39% of NSS trials (36/92).

### Potentially clinically meaningful effects and authors’ conclusions in not statistically significant trials

**Fig. 4:**
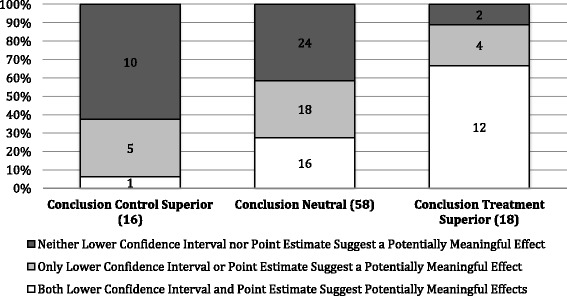
The proportion of lower confidences intervals and/or point estimates that suggest potentially meaningful effects within conclusions from not statistically significant trials. Potentially meaningful lower confidence interval ≤0.75 relative risk; potentially meaningful point estimate ≤0.94 relative risk

Figure 4 shows the findings based on authors’ conclusions and whether the lower confidence intervals and/or point estimates of the primary outcomes included potentially meaningful effects. The lower confidence interval and point estimate included potentially meaningful effects in 67% of trials (12/18) with conclusions that treatment was superior compared to 6% of trials (1/16) with conclusions that control was superior. Neither the lower confidence interval nor point estimate included potentially meaningful effects in 11% of trials (2/18) with conclusions that treatment was superior compared to 63% of trials (10/16) with conclusions that control was superior. The point estimates and lower confidence intervals of neutral authors’ conclusions were distributed relatively evenly.

### Factors associated with authors’ conclusions

Table 2 shows NSS trial abstract conclusions compared to selected study characteristics, including the type of comparator used (placebo or active), funding (industry or public), point estimate (median and threshold), and lower confidence interval (median and threshold). There was no association between conclusions and type of comparator or funding. Both median point estimates and median lower confidence intervals decline as authors’ conclusions change from control superior to neutral to treatment superior (both *p* ≤ 0.006). Additionally, the clinically meaningful thresholds for point estimates and lower confidence intervals were statistically significantly associated with authors’ conclusion (*p* ≤ 0.002). These findings consistently show a similar association for NSS trials: lower point estimates and/or confidence intervals that suggest potentially clinical effects are associated with authors’ concluding that treatment is superior.

**Table 2:** Abstract conclusions of included not statistically significant trials with a superiority design categorized by study characteristics

	Authors’ conclusion in the abstract			*p*-value
	Control superior	Neutral	Treatment superior	
Number of studies	16	58	18	
Comparator				
Placebo/nothing: 49 studies (%)	12 (24)	29 (59)	8 (16)	0.15^a^
Standard/active comparator: 43 studies (%)	4 (9)	29 (67)	10 (23)	
Funding				
Industry: 37 studies (%)	9 (24)	21 (57)	7 (19)	0.14^a^
Mixed: 35 studies (%)	7 (20)	20 (57)	8 (23)	
Public: 20 studies (%)	0	17 (85)	3 (15)	
Point estimate				
Median (interquartile range)	0.99 (0.96–1.09)	0.95 (0.90–1.01)	0.88 (0.83–0.98)	0.006^b^
Point estimate >0.94: 48 studies (%)	14 (29)	29 (60)	5 (10)	0.002^a^
Point estimate ≤0.94: 44 studies (%)	2 (5)	29 (66)	13 (30)	
Lower confidence interval				
Median (interquartile range)	0.84 (0.73–0.89)	0.79 (0.66–0.86)	0.66 (0.57–0.69)	0.005^b^
Confidence interval >0.75: 51 studies (%)	11 (22)	37 (73)	3 (6)	0.001^a^
Confidence interval ≤0.75: 41 studies (%)	5 (12)	21 (51)	15 (37)	

^a^Chi-square

^b^Independent samples median test

### Sensitivity analyses

Table 3 shows the sensitivity analyses. Subgroups of trial size or primary and secondary prevention generally had similar proportions of trials with potentially meaningful effects. The only exception was the trial size subgroup examining the proportion of NSS trials with confidence intervals that suggested potentially meaningful effects: 71% (25/35) for smaller NSS trials with <2000 patient-years versus 28% (16/57) for larger NSS trials with ≥2000 patient-years. The proportion of larger NSS trials with a point estimate and/or confidence interval including potentially meaningful effects was 53% (30/57).

**Table 3:** Sensitivity analysis of not statistically significant randomized controlled trials

Subgroups	Categories	Point estimate Relative risk		Confidence interval Relative risk	
		≤0.94	>0.94	≤0.75	>0.75
Study size (in patient years)	<2000 patient-years (n = 35)	16 (46%)	19 (54%)	25 (71%)	10 (29%)
	≥2000 patient-years (n = 57)	28 (49%)	29 (51%)	16 (28%)	41 (72%)
Primary versus secondary prevention	Primary (n = 13)	5 (38%)	8 (62%)	5 (38%)	8 (62%)
	Secondary (n = 79)	39 (49%)	40 (51%)	36 (46%)	43 (54%)
		≤0.85	>0.85	≤0.65	>0.65
Increase in potentially clinically meaningful thresholds (n = 92)		16 (17%)	76 (83%)	22 (24%)	70 (76%)

Lastly, NSS trials were re-examined using increased potentially clinically meaningful thresholds. The increased thresholds were a relative risk reduction of ≥15% for point estimates and ≥35% for lower confidence intervals. In 15% of NSS trials (14/92) both the increased point estimate and confidence interval included potentially meaningful effects, in 11% (10/92) only one of the two included a potentially meaningful effect, and in 74% (68/92) neither threshold was met.

## Discussion

In 61% of NSS cardiovascular trials, the primary outcome had a confidence interval that included an effect similar to or better than statin therapy (relative risk reduction ≥25%) and/or a point estimate similar to or better than ezetimibe (≥6%). These results suggest that if we were to strictly focus on a dichotomous finding of whether results are SS or NSS, we run the risk of dismissing a treatment in almost two thirds of NSS trials that could potentially have meaningful effects. Furthermore, about one third of NSS trials had even higher probability of potentially clinically meaningful effects because both confidence intervals and point estimates included potentially meaningful effects. In fact, visual inspection of Fig. 2 shows the distribution of the effects is very similar between SS trials favoring treatment and NSS trials when both confidence interval and point estimates include potential meaningful effects. This further suggests that strict adherence to an arbitrary threshold for statistical significance may serve poorly as a judgment of treatment benefit.

Within NSS trials, authors’ conclusions were associated with the potentially meaningful effects in the confidence intervals and point estimates. For example, both the point estimate and confidence intervals included potentially meaningful effects in 67% of NSS trials in which the authors concluded treatment was superior. In contrast, both the point estimate and confidence intervals included potentially meaningful effects in only 6% of NSS in which the authors’ concluded control was superior. Past research suggested that just over half of NSS studies have conclusions that are unjustifiably positive and inconsistent with the results [7]. However, our study suggests that some of these favorable interpretations may relate to potentially meaningful benefits suggested in the confidence intervals and/or point estimates. Given this and the recommendations of CONSORT regarding the presentation of results [1], future research evaluating authors’ interpretations or conclusions of NSS trials should assess trial outcomes beyond statistical significance testing.

Potentially meaningful effects in the point estimates and confidence intervals are not the only factors influencing authors’ conclusions. For example, 28% of NSS trials with a neutral conclusion had both a lower confidence interval and point estimate suggestive of potentially meaningful effects. Perhaps these authors are basing their conclusions solely on statistical significance but it is also possible that other elements of the trial results or intervention play a role: adverse events, costs, and secondary outcomes are all potentially relevant.

Our results were sensitive to two possibly predictable factors. First, trials of smaller size frequently have less precision in the estimate and thus broader confidence intervals. Within our study, this could result in more of the smaller trials having lower confidence intervals crossing a potentially meaningful threshold. This did occur but most of the trials included in this review were large. Therefore, the proportion of NSS trials in which either the point estimate and/or the confidence interval included potentially meaningful effects was only slightly lower in larger trials (having ≥2000 patient-years) than overall (53% versus 61%, respectively). Second, modification of the thresholds of potentially clinically meaningful effects foreseeably reduced the proportion of trials with potentially meaningful effects. The proportion of NSS trials in which either the point estimate and/or the confidence interval included potentially meaningful effects was 61% in our primary analysis but fell to 26% when the relative risk reduction thresholds were increased to ≥15% for point estimates and ≥35% for confidence intervals. However, even with these stricter criteria, a quarter of all NSS cardiovascular trials found potentially meaningful effects.

Despite our findings, it is important not to over-interpret our results and assume that we are suggesting that a 6% relative risk reduction is a meaningful effect in all populations. Nor would we suggest all researchers use these thresholds for sample size estimation and/or extended or repeated studies until these small benefits are entirely ruled out. All interventions, and the trials assessing their clinical value, need to be considered in the boarder context of many relevant factors, including overall risk of the primary outcome, adverse events, costs, inconvenience, and alternative interventions. We hope this paper can draw attention to the need to use confidence intervals and describe potentially meaningful effects. Fortunately, it appears that a number of authors are already doing this. Moreover, we support the advice [19] that authors and evidence-users move away from the dogmatic adherence to hypothesis testing that leads some to believe that a *p*-value of 0.049 means a positive trial and treatment works while a *p*-value of 0.051 means a negative trial and treatment does not work.

There are some notable limitations to our study. First, there are many factors involved in how authors interpret their research but our study focused only on point estimates and confidence intervals of primary outcomes. Second, we focused on cardiovascular trials with hard clinical (MACE) endpoints and so confirmation is required to determine if results would be similar for research in other conditions like chronic obstructive pulmonary disease or infectious disease. Third, our definitions of potentially clinically meaningful effects may be seen as arbitrary or too generous. There is no agreed-on minimal clinically important effect for MACE outcomes so we derived our definition from established therapies although some will certainly feel they are too generous. We used somewhat liberal thresholds because our goal was to determine if results included any “potentially” clinically meaningful effects but we also performed a sensitivity analysis with stricter criteria. While some will see these cut-offs as arbitrary, a goal of this paper is to reflect on the rigid adherence to the 0.05 statistic significance threshold, which itself can be considered arbitrary. Fourth, we used relative margins. The use of relative margins allows for more easy comparison across trials because any assessment of absolute effects must also account for time. Fifth, although we assessed authors’ conclusions by focusing on abstract conclusions, this is a previous method of rating conclusions [12] and abstract conclusion is the most likely location for promotion of results [7]. It should also be noted that the abstract conclusions, like any part of the articles, may have been modified through the peer-review process and editorial recommendations. It is not possible to clarify to what, if any, degree this occurred but we suspect it is small.

## Conclusions

In up to 61% of NSS cardiovascular trials, the primary outcome has a point estimate and/or confidence interval that includes potentially clinically meaningful effects. Furthermore, among the NSS cardiovascular trials, authors’ conclusions were positively associated with point estimates and lower confidence intervals that suggest greater potential effects. In fact, both the point estimates and confidence intervals included potentially meaningful effects in 67% of trials (12/18) in which the authors concluded that treatment was superior, compared to only 6% (1/16) in which authors concluded that control was superior. Given the frequency of NSS cardiovascular trials, it is reassuring that many authors look beyond statistical significance testing and consider the potentially meaningful clinical effects of their results. Additionally, journals and evidence-users should be encouraged, as directed by CONSORT, to consider point estimates and confidence intervals in the context of potentially clinically meaningful effects and not strictly for hypothesis and statistical significance testing.

## Additional file

Additional file 1: Supplementary material: included studies. (DOCX 61 kb)
