# Supplementary material for: Are potentially clinically meaningful benefits misinterpreted in cardiovascular randomized trials? A systematic examination of statistical significance, clinical significance, and authors’ conclusions
Source: BMC Med. 2017 Mar 20;15:58. doi: 10.1186/s12916-017-0821-9 (PMC5357813; doi:10.1186/s12916-017-0821-9)
Supplement: Supplementary file 1 — Supplementary material: included studies. (DOCX 61 kb) [file 12916_2017_821_MOESM1_ESM.docx]

Supplemental Material

Are Potentially Clinically Meaningful Benefits Misinterpreted in Cardiovascular Randomized Trails? A Systematic Examination of Statistical Significance, Clinical Significance and Authors’ Conclusions

Page 2-14,………………………………..…………………………………………………Included Studies

Included Studies

|  | Citation | Statistical Significance Testing |
| --- | --- | --- |
| 1 | Di Biase L, Burkhardt JD, Santangeli P, Mohanty P, Sanchez JE, et al. Periprocedural stroke and bleeding complications in patients undergoing catheter ablation of atrial fibrillation with different anticoagulation management: results from the Role of Coumadin in Preventing Thromboembolism in Atrial Fibrillation (AF) Patients Undergoing Catheter Ablation (COMPARE) randomized trial. Circulation. 2014 Jun 24; 129(25):2638-44. | SS Treatment |
| 2 | De Bruyne B, Pijls NH, Kalesan B, Barbato E, Tonino PA, Piroth Z, et al. Fractional flow reserve-guided PCI versus medical therapy in stable coronary disease. N Engl J Med. 2012 Sep 13;367(11):991-1001. | SS Treatment |
| 3 | Mohr JP, Parides MK, Stapf C, Moquete E, Moy CS, Overbey JR,  et al.  Medical management with or without interventional therapy for unruptured brain arteriovenous malformations (ARUBA): a multicentre, non-blinded, randomised trial. Lancet. 2014 Feb 15;383(9917):614-21. | SS Treatment |
| 4 | Wald DS, Morris JK, Wald NJ, Chase AJ, Edwards RJ, Hughes LO, et al. Randomized trial of preventive angioplasty in myocardial infarction. N Engl J Med. 2013 Sep 19;369(12):1115-23. | SS Treatment |
| 5 | De Bruyne B, Fearon WF, Pijls NH, Barbato E, Tonino P, Piroth Z, et al. Fractional flow reserve-guided PCI for stable coronary artery disease. N Engl J Med. 2014 Sep 25;371(13):1208-17 | SS Treatment |
| 6 | DeBaun MR, Gordon M, McKinstry RC, Noetzel MJ, White DA, Sarnaik SA, et al. Controlled trial of transfusions for silent cerebral infarcts in sickle cell anemia. N Engl J Med. 2014 Aug 21;371(8):699-710. | SS Treatment |
| 7 | Connolly SJ, Eikelboom J, Joyner C, Diener HC, Hart R, Golitsyn S, et al. Apixaban in patients with atrial fibrillation. N Engl J Med. 2011 Mar 3;364(9):806-17. | SS Treatment |
| 8 | Räber L, Kelbæk H, Ostojic M, Baumbach A, Heg D, Tüller D, et al. Effect of biolimus-eluting stents with biodegradable polymer vs bare-metal stents on cardiovascular events among patients with acute myocardial infarction: the COMFORTABLE AMI randomized trial. JAMA. 2012 Aug 22;308(8):777-87. | SS Treatment |
| 9 | Schierbeck LL, Rejnmark L, Tofteng CL, Stilgren L, Eiken P, Mosekilde L, et al. Effect of hormone replacement therapy on cardiovascular events in recently postmenopausal women: randomised trial. BMJ. 2012 Oct 9;345:e6409. | SS Treatment |
| 10 | Zannad F, McMurray JJ, Krum H, van Veldhuisen DJ, Swedberg K, Shi H, et al. Eplerenone in patients with systolic heart failure and mild symptoms. N Engl J Med. 2011 Jan 6;364(1):11-21. | SS Treatment |
| 11 | Mehilli J, Pache J, Abdel-Wahab M, Schulz S, Byrne RA, et al. Drug-eluting versus bare-metal stents in saphenous vein graft lesions (ISAR-CABG): a randomised controlled superiority trial. Lancet. 2011 Sept 17;378:1071-78. | SS Treatment |
| 12 | Wang Y, Wang Y, Zhao X, Liu L, Wang D, Wang C, et al. Clopidogrel with aspirin in acute minor stroke or transient ischemic attack.N Engl J Med. 2013 Jul 4;369(1):11-9. | SS Treatment |
| 13 | Kedhi E, Joesoef KS, McFadden E, Wassing J, van Mieghem C, et al. Second-generation everolimus-eluting and paclitaxel-eluting stents in real-life practice (COMPARE): a randomised trial. Lancet. 2010 Jan 16;375(9711):201-9. | SS Treatment |
| 14 | Estruch R, Ros E, Salas-Salvado J, Covas MI, Corella D, Aros F, et al. Primary prevention of cardiovascular disease with a Mediterranean diet. N Engl J Med. 2013 Apr 4;368(14):1279-90. | SS Treatment |
| 15 | Estruch R, Ros E, Salas-Salvado J, Covas MI, Corella D, Aros F, et al. Primary prevention of cardiovascular disease with a Mediterranean diet. N Engl J Med. 2013 Apr 4;368(14):1279-90. | SS Treatment |
| 16 | Mauri L, Kereiakes DJ, Yeh RW, Driscoll-Shempp P, Cutlip DE, Steg PG, et al. Twelve or 30 months of dual antiplatelet therapy after drug-eluting stents. N Engl J Med. 2014 Dec 4;371(23):2155-66 | SS Treatment |
| 17 | Farkouh ME, Domanski M, Sleeper LA, Siami FS, Dangas G, Mack M, et al. Strategies for multivessel revascularization in patients with diabetes. N Engl J Med. 2012 Dec 20;367(25):2375-84. | SS Treatment |
| 18 | Bhatt DL, Stone GW, Mahaffey KW, Gibson CM, Steg PG, Hamm CW, et al; CHAMPION PHOENIX Investigators. Effect of platelet inhibition with cangrelor during PCI on ischemic events. N Engl J Med. 2013 Apr 4;368(14):1303-13. | SS Treatment |
| 19 | McMurray JJ, Packer M, Desai AS, Gong J, Lefkowitz MP, Rizkala AR, et al. Angiotensin-neprilysin inhibition versus enalapril in heart failure. N Engl J Med. 2014 Sep 11;371(11):993-1004. | SS Treatment |
| 20 | Swedberg K, Komajda M, Bšhm M, Borer JS, Ford I, Dubost-Brama A, et al. Ivabradine and outcomes in chronic heart failure (SHIFT): a randomised placebo-controlled study. Lancet. 2010 Sep 11;376(9744):875-85. | SS Treatment |
| 21 | Lamas GA, Goertz C, Boineau R, Mark DB, Rozema T, Nahin RL, et al. Effect of disodium EDTA chelation regimen on cardiovascular events in patients with previous myocardial infarction: the TACT randomized trial. JAMA. 2013 Mar 27;309(12):1241-50. | SS Treatment |
| 22 | Baigent C, Landray MJ, Reith C, Emberson J, Wheeler DC, Tomson C, et al. The effects of lowering LDL cholesterol with simvastatin plus ezetimibe in patients with chronic kidney disease (Study of Heart and Renal Protection): a randomised placebo-controlled trial. Lancet. 2011 Jun 25;377(9784):2181-92. | SS Treatment |
| 23 | Mega JL, Braunwald E, Wiviott SD, Bassand JP, Bhatt DL, Bode C, et al. Rivaroxaban in patients with a recent acute coronary syndrome. N Engl J Med. 2012 Jan 5;366(1):9-19. | SS Treatment |
| 24 | Cannon CP, Harrington RA, James S, Ardissino D, Becker RC, Emanuelsson H, et al. Comparison of ticagrelor with clopidogrel in patients with a planned invasive strategy for acute coronary syndromes (PLATO): a randomised double-blind study. Lancet. 2010 Jan 23;375(9711):283-93. | SS Treatment |
| 25 | Morrow DA, Braunwald E, Bonaca MP, Ameriso SF, Dalby AJ, Fish MP, et al. Vorapaxar in the secondary prevention of atherothrombotic events. N Engl J Med. 2012 Apr 12;366(15):1404-13. | SS Treatment |
| 26 | Choudhry NK, Avorn J, Glynn RJ, Antman EM, Schneeweiss S, Toscano M, et al. Full coverage for preventive medications after myocardial infarction. N Engl J Med. 2011 Dec 1;365(22):2088-97. | SS Treatment |
| 27 | Kaczorowski J, Chambers LW, Dolovich L, Paterson JM, Karwalajtys T, Gierman T, et al. Improving cardiovascular health at population level: 39 community cluster randomised trial of Cardiovascular Health Awareness Program (CHAP). BMJ. 2011 Feb 7;342:d442. | SS Treatment |
| 28 | HPS2-THRIVE Collaborative Group, Landray MJ, Haynes R, Hopewell JC, Parish S, Aung T, et al. Effects of extended-release niacin with laropiprant in high-risk patients. N Engl J Med. 2014 Jul 17;371(3):203-12. | NSS |
| 29 | de Zeeuw D, Akizawa T, Audhya P, Bakris GL, Chin M, Christ-Schmidt H, et al. Bardoxolone methyl in type 2 diabetes and stage 4 chronic kidney disease. N Engl J Med. 2013 Dec 26;369(26):2492-503. | NSS |
| 30 | SPS3 Investigators, Benavente OR, Hart RG, McClure LA, Szychowski JM, Coffey CS, Pearce LA. Effects of clopidogrel added to aspirin in patients with recent lacunar stroke. N Engl J Med. 2012 Aug 30;367(9):817-25. | NSS |
| 31 | Kastrati A, Neumann FJ, Schulz S, Massberg S, Byrne RA, et al. Abciximab and heparin versus bivalirudin for non-ST-elevation myocardial infarction. N Engl J Med. 2011 Nov 24;365(21):1980-9. | NSS |
| 32 | Tricoci P, Huang Z, Held C, Moliterno DJ, Armstrong PW, et al., Thrombin-receptor antagonist vorapaxar in acute coronary syndromes. N Engl J Med. 2012 Jan 5;366(1):20-33. | NSS |
| 33 | Alexander JH, Lopes RD, James S, Kilaru R, He Y, Mohan P, et al. Apixaban with antiplatelet therapy after acute coronary syndrome. N Engl J Med. 2011 Aug 25;365(8):699-708. | NSS |
| 34 | Collet JP, Silvain J, Barthelemy O, Range G, Cayla G, et al. Dual-antiplatelet treatment beyond 1 year after drug-eluting stent implantation (ARCTIC-Interruption): a randomised trial. Lancet. 2014. 384:1577-85. | NSS |
| 35 | Topol EJ, Bousser MG, Fox KA, Creager MA, Despres JP, Easton JD, et al. Rimonabant for prevention of cardiovascular events (CRESCENDO): a randomised, multicentre, placebo-controlled trial. Lancet. 2010 Aug 14;376(9740):517-23. | NSS |
| 36 | Nicholls SJ, Kastelein JJ, Schwartz GG, Bash D, Rosenson RS, Cavender MA, et al. Varespladib and cardiovascular events in patients with an acute coronary syndrome: the VISTA-16 randomized clinical trial. JAMA. 2014 Jan 15;311(3):252-62. | NSS |
| 37 | Steg PG, Mehta SR, Pollack CV Jr, Bode C, Cohen M, French WJ, et al. Anticoagulation with otamixaban and ischemic events in non-ST-segment elevation acute coronary syndromes: the TAO randomized clinical trial. JAMA. 2013 Sep 18;310(11):1145-55. | NSS |
| 38 | Devereaux PJ, Mrkobrada M, Sessler DI, Leslie K, Alonso-Coello P, Kurz A, et al. Aspirin in patients undergoing noncardiac surgery. N Engl J Med. 2014 Apr 17;370(16):1494-503. | NSS |
| 39 | Devereaux PJ, Sessler DI, Leslie K, Kurz A, Mrkobrada M, Alonso-Coello P, et al. Clonidine in patients undergoing noncardiac surgery. N Engl J Med. 2014 Apr 17;370(16):1504-13 | NSS |
| 40 | Montalescot G, Bolognese L, Dudek D, Goldstein P, Hamm C, Tanguay JF, et al. Pretreatment with prasugrel in non-ST-segment elevation acute coronary syndromes. N Engl J Med. 2013 Sep 12;369(11):999-1010. | NSS |
| 41 | Parving HH, Brenner BM, McMurray JJ, de Zeeuw D, Haffner SM, Solomon SD, et al. Cardiorenal end points in a trial of aliskiren for type 2 diabetes. N Engl J Med. 2012 Dec 6;367(23):2204-13 | NSS |
| 42 | Sandset EC, Bath PM, Boysen G, Jatuzis D, K›rv J, LŸders S, et al. The angiotensin-receptor blocker candesartan for treatment of acute stroke (SCAST): a randomised, placebo-controlled, double-blind trial. Lancet. 2011 Feb 26;377(9767):741-50. | NSS |
| 43 | Shaw LJ, Mieres JH, Hendel RH, Boden WE, Gulati M, et al. Comparative effectiveness of exercise electrocardiography with or without myocardial perfusion single photon emission computed tomography in women with suspected coronary artery disease: results from the What Is the Optimal Method for Ischemia Evaluation in Women (WOMEN) trial. Circulation. 2011 Sep 13; 124(11):1239-49. | NSS |
| 44 | Fox K, Ford I, Steg PG, Tardif JC, Tendera M, Ferrari R; SIGNIFY Investigators. Ivabradine in stable coronary artery disease without clinical heart failure.N Engl J Med. 2014 Sep 18;371(12):1091-9. | NSS |
| 45 | Pitt B, Pfeffer MA, Assmann SF, Boineau R, Anand IS, Claggett B, et al. Spironolactone for heart failure with preserved ejection fraction. N Engl J Med. 2014 Apr 10;370(15):1383-92. | NSS |
| 46 | STABILITY Investigators, White HD, Held C, Stewart R, Tarka E, Brown R, et al. Darapladib for preventing ischemic events in stable coronary heart disease.N Engl J Med. 2014 May 1;370(18):1702-11 | NSS |
| 47 | Cooper CJ, Murphy TP, Cutlip DE, Jamerson K, Henrich W, Reid DM, et al. Stenting and medical therapy for atherosclerotic renal-artery stenosis. N Engl J Med. 2014 Jan 2;370(1):13-22. | NSS |
| 48 | Look AHEAD Research Group, Wing RR, Bolin P, Brancati FL, Bray GA, Clark JM, Coday M, et al. Cardiovascular effects of intensive lifestyle intervention in type 2 diabetes. N Engl J Med. 2013 Jul 11;369(2):145-54. | NSS |
| 49 | Risk and Prevention Study Collaborative Group, Roncaglioni MC, Tombesi M, Avanzini F, Barlera S, Caimi V, Longoni P, et al. n-3 fatty acids in patients with multiple cardiovascular risk factors. N Engl J Med. 2013 May 9;368(19):1800-8. | NSS |
| 50 | Meier B, Kalesan B, Mattle HP, Khattab AA, Hildick-Smith D, Dudek D, et al. Percutaneous closure of patent foramen ovale in cryptogenic embolism. N Engl J Med. 2013 Mar 21;368(12):1083-91. | NSS |
| 51 | Lamy A, Devereaux PJ, Prabhakaran D, Taggart DP, Hu S, Paolasso E, et al. Effects of off-pump and on-pump coronary-artery bypass grafting at 1 year. N Engl J Med. 2013 Mar 28;368(13):1179-88. | NSS |
| 52 | Diegeler A, Bšrgermann J, Kappert U, Breuer M, Bšning A, Ursulescu A,et al. Off-pump versus on-pump coronary-artery bypass grafting in elderly patients. N Engl J Med. 2013 Mar 28;368(13):1189-98. | NSS |
| 53 | Collet JP, Cuisset T, RangŽ G, Cayla G, Elhadad S, Pouillot C, et al. Bedside monitoring to adjust antiplatelet therapy for coronary stenting. N Engl J Med. 2012 Nov 29;367(22):2100-9. | NSS |
| 54 | EVOLVE Trial Investigators, Chertow GM, Block GA, Correa-Rotter R, Drüeke TB, Floege J, Goodman WG, et al. Effect of cinacalcet on cardiovascular disease in patients undergoing dialysis. N Engl J Med. 2012 Dec 27;367(26):2482-94. | NSS |
| 55 | Roe MT, Armstrong PW, Fox KAA, White HD, Prabhakaran D, et al. Prasugrel versus clopidogrel for acute coronary syndromes without revascularization. N Engl J Med. 2012 Oct 4;367(14):1297-309. | NSS |
| 56 | ORIGIN Trial Investigators, Gerstein HC, Bosch J, Dagenais GR, Díaz R, Jung H, Maggioni AP, et al. Basal insulin and cardiovascular and other outcomes in dysglycemia. N Engl J Med. 2012 Jul 26;367(4):319-28. | NSS |
| 57 | ORIGIN Trial Investigators, Bosch J, Gerstein HC, Dagenais GR, Díaz R, Dyal L, Jung H et al. n-3 fatty acids and cardiovascular outcomes in patients with dysglycemia. N Engl J Med. 2012 Jul 26;367(4):309-18. | NSS |
| 58 | Homma S, Thompson JL, Pullicino PM, Levin B, Freudenberger RS, Teerlink JR, et al. Warfarin and aspirin in patients with heart failure and sinus rhythm. N Engl J Med. 2012 May 17;366(20):1859-69. | NSS |
| 59 | Lamy A, Devereaux PJ, Prabhakaran D, Taggart DP, Hu S, Paolasso E, et al. Off-pump or on-pump coronary-artery bypass grafting at 30 days. N Engl J Med. 2012 Apr 19;366(16):1489-97. | NSS |
| 60 | Furlan AJ, Reisman M, Massaro J, Mauri L, Adams H, Albers GW, et al. Closure or medical therapy for cryptogenic stroke with patent foramen ovale. N Engl J Med. 2012;366(11):991-9. | NSS |
| 61 | AIM-HIGH Investigators, Boden WE, Probstfield JL, Anderson T, Chaitman BR, Desvignes-Nickens P, Koprowicz K, et al. Niacin in patients with low HDL cholesterol levels receiving intensive statin therapy. N Engl J Med. 2011 Dec 15;365(24):2255-67. | NSS |
| 62 | ACTIVE I Investigators, Yusuf S, Healey JS, Pogue J, Chrolavicius S, Flather M, Hart RG, et al. Irbesartan in patients with atrial fibrillation. N Engl J Med. 2011 Mar 10;364(10):928-38. | NSS |
| 63 | Kaiser C, Galatius S, Erne P, Eberli F, Alber H, Rickli H, et al. Drug-eluting versus bare-metal stents in large coronary arteries. N Engl J Med. 2010 Dec 9;363(24):2310-9. | NSS |
| 64 | Kaiser C, Galatius S, Erne P, Eberli F, Alber H, Rickli H, et al. Drug-eluting versus bare-metal stents in large coronary arteries. N Engl J Med. 2010 Dec 9;363(24):2310-9. | NSS |
| 65 | Matchar DB, Jacobson A, Dolor R, Edson R, Uyeda L, Phibbs CS, et al. Effect of home testing of international normalized ratio on clinical events. N Engl J Med. 2010 Oct 21;363(17):1608-20. | NSS |
| 66 | Kromhout D, Giltay EJ, Geleijnse JM; Alpha Omega Trial Group. n-3 fatty acids and cardiovascular events after myocardial infarction. N Engl J Med. 2010 Nov 18;363(21):2015-26. | NSS |
| 67 | Kromhout D, Giltay EJ, Geleijnse JM; Alpha Omega Trial Group. n-3 fatty acids and cardiovascular events after myocardial infarction. N Engl J Med. 2010 Nov 18;363(21):2015-26. | NSS |
| 68 | CURRENT-OASIS 7 Investigators, Mehta SR, Bassand JP, Chrolavicius S, Diaz R, Eikelboom JW, Fox KA, et al. Dose comparisons of clopidogrel and aspirin in acute coronary syndromes. N Engl J Med. 2010 Sep 2;363(10):930-42. | NSS |
| 69 | CURRENT-OASIS 7 Investigators, Mehta SR, Bassand JP, Chrolavicius S, Diaz R, Eikelboom JW, Fox KA, et al. Dose comparisons of clopidogrel and aspirin in acute coronary syndromes. N Engl J Med. 2010 Sep 2;363(10):930-42. | NSS |
| 70 | Brott TG, Hobson RW 2nd, Howard G, Roubin GS, Clark WM, Brooks W, et al. Stenting versus endarterectomy for treatment of carotid-artery stenosis. N Engl J Med. 2010 Jul 1;363(1):11-23. | NSS |
| 71 | Park SJ, Park DW, Kim YH, Kang SJ, Lee SW, Lee CW, et al. Duration of dual antiplatelet therapy after implantation of drug-eluting stents. N Engl J Med. 2010 Apr 15;362(15):1374-82. | NSS |
| 72 | ACCORD Study Group, Ginsberg HN, Elam MB, Lovato LC, Crouse JR 3rd, Leiter LA, Linz P, et al. Effects of combination lipid therapy in type 2 diabetes mellitus. N Engl J Med. 2010 Apr 29;362(17):1563-74. | NSS |
| 73 | NAVIGATOR Study Group, Holman RR, Haffner SM, McMurray JJ, Bethel MA, Holzhauer B, Hua TA, et al. Effect of nateglinide on the incidence of diabetes and cardiovascular events. N Engl J Med. 2010 Apr 22;362(16):1463-76. | NSS |
| 74 | ACCORD Study Group, Cushman WC, Evans GW, Byington RP, Goff DC Jr, Grimm RH Jr, Cutler JA, et al. Effects of intensive blood-pressure control in type 2 diabetes mellitus. N Engl J Med. 2010 Apr 29;362(17):1575-85. | NSS |
| 75 | Maeng M, Tilsted HH, Jensen LO, Krusell LR, Kaltoft A, Kelb¾k H, et al. Differential clinical outcomes after 1 year versus 5 years in a randomised comparison of zotarolimus-eluting and sirolimus-eluting coronary stents (the SORT OUT III study): a multicentre, open-label, randomised superiority trial. Lancet. 2014 Jun 14;383(9934):2047-56. | NSS |
| 76 | Muhlestein JB, Lappé DL, Lima JA, Rosen BD, May HT, Knight S, et al. Effect of screening for coronary artery disease using CT angiography on mortality and cardiac events in high-risk patients with diabetes: the FACTOR-64 randomized clinical trial. JAMA. 2014 Dec 3;312(21):2234-43. | NSS |
| 77 | Ikeda Y, Shimada K, Teramoto T, Uchiyama S, Yamazaki T, Oikawa S, et al. Low-dose aspirin for primary prevention of cardiovascular events in Japanese patients 60 years or older with atherosclerotic risk factors: a randomized clinical trial. JAMA. 2014 Dec 17;312(23):2510-20. | NSS |
| 78 | O'Donoghue M, Braunwald E, White HD, Steen DL, Lukas MA, et al. Effect of darapladib on major coronary events after an acute coronary syndrome: the SOLID-TIMI 52 randomized clinical trial.. JAMA. 2014 Sept 10;312(10):1006-15. | NSS |
| 79 | Gheorghiade M, Bohm M, Greene SJ, Fonarow GC, Lewis EF, et al. Effect of aliskiren on postdischarge mortality and heart failure readmissions among patients hospitalized for heart failure: the ASTRONAUT randomized trial. JAMA. 2013 Mar 20;309(11):1125-35. | NSS |
| 80 | Dieleman JM, Nierich AP, Rosseel PM, van der Maaten JM, Hofland J, Diephuis JC, et al. Intraoperative high-dose dexamethasone for cardiac surgery: a randomized controlled trial. JAMA. 2012 Nov 7;308(17):1761-7. | NSS |
| 81 | Sesso HD, Christen WG, Bubes V, Smith JP, MacFadyen J, Schvartz M, et al. Multivitamins in the prevention of cardiovascular disease in men: the Physicians' Health Study II randomized controlled trial. JAMA. 2012 Nov 7;308(17):1751-60. | NSS |
| 82 | Newman MF, Ferguson TB, White JA, Ambrosio G, Koglin J, Nussmeier NA, et al. Effect of adenosine-regulating agent acadesine on morbidity and mortality associated with coronary artery bypass grafting: the RED-CABG randomized controlled trial. JAMA. 2012 Jul 11;308(2):157-64. | NSS |
| 83 | Barbé F, Durán-Cantolla J, Sánchez-de-la-Torre M, Martínez-Alonso M, Carmona C, Barceló A, et al. Effect of continuous positive airway pressure on the incidence of hypertension and cardiovascular events in nonsleepy patients with obstructive sleep apnea: a randomized controlled trial. JAMA. 2012 May 23;307(20):2161-8. | NSS |
| 84 | Powers WJ, Clarke WR, Grubb RL Jr, Videen TO, Adams HP Jr, Derdeyn CP; COSS Investigators. Extracranial-intracranial bypass surgery for stroke prevention in hemodynamic cerebral ischemia: the Carotid Occlusion Surgery Study randomized trial. JAMA. 2011 Nov 9;306(18):1983-92. | NSS |
| 85 | Price MJ, Berger PB, Teirstein PS, Tanguay JF, Angiolillo DJ, Spriggs D, et al. Standard- vs high-dose clopidogrel based on platelet function testing after percutaneous coronary intervention: the GRAVITAS randomized trial. JAMA. 2011 Mar 16;305(11):1097-105. | NSS |
| 86 | Perera D, Stables R, Thomas M, Booth J, Pitt M, Blackman D, et al. Elective intra-aortic balloon counterpulsation during high-risk percutaneous coronary intervention: a randomized controlled trial. JAMA. 2010 Aug 25;304(8):867-74. | NSS |
| 87 | Study of the Effectiveness of Additional Reductions in Cholesterol and Homocysteine (SEARCH) Collaborative Group, Armitage JM, Bowman L, Clarke RJ, Wallendszus K, Bulbulia R, Rahimi K, et al. Effects of homocysteine-lowering with folic acid plus vitamin B12 vs placebo on mortality and major morbidity in myocardial infarction survivors: a randomized trial. AMA. 2010 Jun 23;303(24):2486-94. | NSS |
| 88 | Fowkes FG, Price JF, Stewart MC, Butcher I, Leng GC, Pell AC, et al. Aspirin for prevention of cardiovascular events in a general population screened for a low ankle brachial index: a randomized controlled trial. JAMA. 2010 Mar 3;303(9):841-8. | NSS |
| 89 | Dracup K, Moser DK, Pelter MM, Nesbitt TS, Southard J, Paul SM, Robinson S, Cooper LS . Randomized, controlled trial to improve self-care in patients with heart failure living in rural areas. Circulation. 2014 Jul 15; 130(3):256-64. | NSS |
| 90 | Dracup K, Moser DK, Pelter MM, Nesbitt TS, Southard J, Paul SM, Robinson S, Cooper LS . Randomized, controlled trial to improve self-care in patients with heart failure living in rural areas. Circulation. 2014 Jul 15; 130(3):256-64. | NSS |
| 91 | Lee CW, Ahn JM, Park DW, Kang SJ, Lee SW, et al. .Optimal duration of dual antiplatelet therapy after drug-eluting stent implantation: a randomized, controlled trial. Circulation. 2014 Jan 21; 129(3):304-12. | NSS |
| 92 | Lurati Buse GA, Schumacher P, Seeberger E, Studer W, Schuman RM, Fassl J, Kasper J, Filipovic M, Bolliger D, Seeberger MD.Randomized comparison of sevoflurane versus propofol to reduce perioperative myocardial ischemia in patients undergoing noncardiac surgery. Circulation. 2012 Dec 4; 126(23):2696-704. | NSS |
| 93 | Valgimigli M, Campo G, Monti M, Vranckx P, Percoco G, et al. Short- versus long-term duration of dual-antiplatelet therapy after coronary stenting: a randomized multicenter trial. Circulation. 2012 Apr 24; 125(16):2015-26. | NSS |
| 94 | Bostom AG, Carpenter MA, Kusek JW, Levey AS, Hunsicker L, et al. Homocysteine-lowering and cardiovascular disease outcomes in kidney transplant recipients: primary results from the Folic Acid for Vascular Outcome Reduction in Transplantation trial. Circulation. 2011 Apr 26; 123(16):1763-70. | NSS |
| 95 | NiemelÃ¤ M, Kervinen K, Erglis A, Holm NR, Maeng M, et al. Randomized comparison of final kissing balloon dilatation versus no final kissing balloon dilatation in patients with coronary bifurcation lesions treated with main vessel stenting: the Nordic-Baltic Bifurcation Study III. Circulation. 2011 Jan 4; 123(1):79-86. | NSS |
| 96 | Rauch B, Schiele R, Schneider S, Diller F, Victor N, Gohlke H, et al. OMEGA, a randomized, placebo-controlled trial to test the effect of highly purified omega-3 fatty acids on top of modern guideline-adjusted therapy after myocardial infarction. Circulation. 2010 Nov 23; 122(21):2152-9. | NSS |
| 97 | MÃ¸ller CH, Perko MJ, Lund JT, Andersen LW, Kelbaek H, Madsen JK, Winkel P, Gluud C, SteinbrÃ¼chel DA. No major differences in 30-day outcomes in high-risk patients randomized to off-pump versus on-pump coronary bypass surgery: the best bypass surgery trial. Circulation. 2010 Feb 2; 121(4):498-504. | NSS |
| 98 | Jørgensen T, Jacobsen RK, Toft U, Aadahl M, Glümer C, Pisinger C. Effect of screening and lifestyle counselling on incidence of ischaemic heart disease in general population: Inter99 randomised trial. BMJ. 2014 Jun 9;348:g3617. | NSS |
| 99 | Galan P, Kesse-Guyot E, Czernichow S, Briancon S, Blacher J, Hercberg S; et al. Effects of B vitamins and omega 3 fatty acids on cardiovascular diseases: a randomised placebo controlled trial. BMJ. 2010 Nov 29;341:c6273. | NSS |
| 100 | Galan P, Kesse-Guyot E, Czernichow S, Briancon S, Blacher J, Hercberg S; et al. Effects of B vitamins and omega 3 fatty acids on cardiovascular diseases: a randomised placebo controlled trial. BMJ. 2010 Nov 29;341:c6273. | NSS |
| 101 | Lamas GA, Boineau R, Goertz C, Mark DB, Rosenberg Y, Stylianou M, et al. Oral high-dose multivitamins and minerals after myocardial infarction: a randomized trial. Ann Intern Med. 2013 Dec 17;159(12):797-805. | NSS |
| 102 | Montalescot G, Zeymer U, Silvain J, Boulanger B, Cohen M, Goldstein P, et al. Intravenous enoxaparin or unfractionated heparin in primary percutaneous coronary intervention for ST-elevation myocardial infarction: the international randomised open-label ATOLL trial. Lancet. 2011 Aug 20;378(9792):693-703. | NSS |
| 103 | Carroll JD, Saver JL, Thaler DE, Smalling RW, Berry S, MacDonald LA, et al. Closure of patent foramen ovale versus medical therapy after cryptogenic stroke. N Engl J Med. 2013 Mar 21;368(12):1092-100. | NSS |
| 104 | Armstrong PW, Gershlick AH, Goldstein P, Wilcox R, Danays T, Lambert Y, et al. Fibrinolysis or primary PCI in ST-segment elevation myocardial infarction. N Engl J Med. 2013 Apr 11;368(15):1379-87. | NSS |
| 105 | Schwartz GG, Olsson AG, Abt M, Ballantyne CM, Barter PJ, Brumm J, et al. Effects of dalcetrapib in patients with a recent acute coronary syndrome. N Engl J Med. 2012 Nov 29;367(22):2089-99. | NSS |
| 106 | Bhatt DL, Cryer BL, Contant CF, Cohen M, Lanas A, Schnitzer TJ, et al. Clopidogrel with or without omeprazole in coronary artery disease. N Engl J Med. 2010 Nov 11;363(20):1909-17. | NSS |
| 107 | NAVIGATOR Study Group, McMurray JJ, Holman RR, Haffner SM, Bethel MA, Holzhauer B, Hua TA, et al. Effect of valsartan on the incidence of diabetes and cardiovascular events. N Engl J Med. 2010 Apr 22;362(16):1477-90. | NSS |
| 108 | Newby LK, Marber MS, Melloni C, Sarov-Blat L, Aberle LH, Aylward PE, et al. Losmapimod, a novel p38 mitogen-activated protein kinase inhibitor, in non-ST-segment elevation myocardial infarction: a randomised phase 2 trial. Lancet. 2014 Sep 27;384(9949):1187-95. | NSS |
| 109 | Benavente OR, Coffey CS, Conwit R, Hart RG, McClure LA, Pearce LA et al. Blood-pressure targets in patients with recent lacunar stroke: the SPS3 randomised trial. Lancet. 2013 Aug 10;382(9891):507-15. | NSS |
| 110 | Sabate M, Cequier A, I–iguez A, Serra A, Hernandez-Antolin R, Mainar V, et al. Everolimus-eluting stent versus bare-metal stent in ST-segment elevation myocardial infarction (EXAMINATION): 1 year results of a randomised controlled trial. Lancet. 2012 Oct 27;380(9852):1482-90. | NSS |
| 111 | Thiele H, Wšhrle J, Hambrecht R, Rittger H, Birkemeyer R, Lauer B, et al. Intracoronary versus intravenous bolus abciximab during primary percutaneous coronary intervention in patients with acute ST-elevation myocardial infarction: a randomised trial. Lancet. 2012 Mar 10;379(9819):923-31. | NSS |
| 112 | Griffin SJ, Borch-Johnsen K, Davies MJ, Khunti K, Rutten GEHM, et al. Effect of early intensive multifactorial therapy on 5-year cardiovascular outcomes in individuals with type 2 diabetes detected by screening (ADDITION-Europe): a cluster-randomised trial. Lancet. 2011 Jul 9;378(9807):156-67. | NSS |
| 113 | Jolly SS, Yusuf S, Cairns J, NiemelŠ K, Xavier D, Widimsky P, et al. Radial versus femoral access for coronary angiography and intervention in patients with acute coronary syndromes (RIVAL): a randomised, parallel group, multicentre trial. Lancet. 2011 Apr 23;377(9775):1409-20. | NSS |
| 114 | Study of the Effectiveness of Additional Reductions in Cholesterol and Homocysteine (SEARCH) Collaborative Group, Armitage J, Bowman L, Wallendszus K, Bulbulia R, Rahimi K, Haynes R, et al. Intensive lowering of LDL cholesterol with 80 mg versus 20 mg simvastatin daily in 12,064 survivors of myocardial infarction: a double-blind randomised trial. Lancet. 2010 Nov 13;376(9753):1658-69. | NSS |
| 115 | Halliday A, Harrison M, Hayter E, Kong X, Mansfield A, et al. 10-year stroke prevention after successful carotid endarterectomy for asymptomatic stenosis (ACST-1): a multicentre randomised trial. Lancet. 2010 Sept 25;376:1074-84. | NSS |
| 116 | Heldman AW, DiFede DL, Fishman JE, Zambrano JP, Trachtenberg BH, Karantalis V, et al. Transendocardial mesenchymal stem cells and mononuclear bone marrow cells for ischemic cardiomyopathy: the TAC-HFT randomized trial. JAMA. 2014 Jan 1;311(1):62-73. | NSS |
| 117 | Heldman AW, DiFede DL, Fishman JE, Zambrano JP, Trachtenberg BH, Karantalis V, et al. Transendocardial mesenchymal stem cells and mononuclear bone marrow cells for ischemic cardiomyopathy: the TAC-HFT randomized trial. JAMA. 2014 Jan 1;311(1):62-73. | NSS |
| 118 | Selker HP, Beshansky JR, Sheehan PR, Massaro JM, Griffith JL, D'Agostino RB, et al. Out-of-hospital administration of intravenous glucose-insulin-potassium in patients with suspected acute coronary syndromes: the IMMEDIATE randomized controlled trial. JAMA. 2012 May 9;307(18):1925-33. | NSS |
| 119 | O'Neill WW, Kleiman NS, Moses J, Henriques JP, Dixon S, Massaro J, Palacios I, Maini B, Mulukutla S, DzavÃ­k V, Popma J, Douglas PS, Ohman M.A prospective, randomized clinical trial of hemodynamic support with Impella 2.5 versus intra-aortic balloon pump in patients undergoing high-risk percutaneous coronary intervention: the PROTECT II study. Circulation. 2012 Oct 2; 126(14):1717-27. | NSS |
| 120 | James WP, Caterson ID, Coutinho W, Finer N, Van Gaal LF, Maggioni AP, et al. Effect of sibutramine on cardiovascular outcomes in overweight and obese subjects. N Engl J Med. 2010 Sep 2;363(10):905-17. | SS Control |
| 121 | Shahzad A, Kemp I, Mars C, Wilson K, Roome C, Cooper R,  et al. Unfractionated heparin versus bivalirudin in primary percutaneous coronary intervention (HEAT-PPCI): an open-label, single centre, randomised controlled trial.  Lancet. 2014 Nov 22;384(9957):1849-58. | SS Control |
| 122 | Derdeyn CP, Chimowitz MI, Lynn MJ, Fiorella D, Turan TN, Janis Alexandrov AV, et al. Aggressive medical treatment with or without stenting in high-risk patients with intracranial artery stenosis (SAMMPRIS): the final results of a randomised trial. Lancet. 2014 Jan 25;383(9914):333-41. | SS Control |
| 123 | International Carotid Stenting Study investigators, Ederle J, Dobson J, Featherstone RL, Bonati LH, van der Worp HB, de Borst GJ, et al. Carotid artery stenting compared with endarterectomy in patients with symptomatic carotid stenosis (International Carotid Stenting Study): an interim analysis of a randomised controlled trial. Lancet. 2010 Mar 20;375(9719):985-97. | SS Control |
| 124 | Chimowitz MI, Lynn MJ, Derdeyn CP, Turan TN, Fiorella D, Lane BF,et al. Stenting versus aggressive medical therapy for intracranial arterial stenosis. N Engl J Med. 2011 Sep 15;365(11):993-1003. | SS Control |
| 125 | Hildick-Smith D, de Belder AJ, Cooter N, Curzen NP, Clayton TC, Oldroyd KG, Bennett L, Holmberg S, Cotton JM, Glennon PE, Thomas MR, Maccarthy PA, Baumbach A, Mulvihill NT, Henderson RA, Redwood SR, Starkey IR, Stables RH.Randomized trial of simple versus complex drug-eluting stenting for bifurcation lesions: the British Bifurcation Coronary Study: old, new, and evolving strategies. Circulation. 2010 Mar 16; 121(10):1235-43. | SS Control |
| 126 | Rasmussen K, Maeng M, Kaltoft A, Thayssen P, Kelbaek H, Tilsted HH, et al. Efficacy and safety of zotarolimus-eluting and sirolimus-eluting coronary stents in routine clinical care (SORT OUT III): a randomised controlled superiority trial. Lancet. 2010 Mar 27;375(9720):1090-9. | SS Control |
| 127 | Connolly SJ, Camm AJ, Halperin JL, Joyner C, Alings M, Amerena J, et al. Dronedarone in high-risk permanent atrial fibrillation. N Engl J Med. 2011 Dec 15;365(24):2268-76. | SS Control |
